# p38γ and p38δ support CD4^+^ T cell responses and T cell-dependent humoral immunity

**DOI:** 10.3389/fimmu.2026.1927358

**Published:** 2026-08-26

**Authors:** Diego González-Romero, Ester Díaz-Mora, Pilar Fajardo, Juan José Sanz-Ezquerro, Yolanda R. Carrasco, Ana Cuenda

**Affiliations:** 1 Department of Immunology and Oncology, Centro Nacional de Biotecnología (CNB)/CSIC, Madrid, Spain; 2 Department of Cellular and Molecular Biology, CNB/CSIC, Madrid, Spain; 3 B Lymphocyte Dynamics, Department of Immunology and Oncology, CNB/CSIC, Madrid, Spain

**Keywords:** CD4, humoral immunity, p38γ MAPK, p38δ MAPK, T follicular helper cells (TFH)

## Abstract

p38γ and p38δ (p38γ/p38δ) have emerged as important regulators of immune function; however, their specific contribution to adaptive humoral responses remains poorly understood. Based on previous evidence that global p38γ/p38δ deficiency impairs antibody production, we investigated the T cell-intrinsic role of these kinases using conditional knockout mouse models. T cell-specific combined deletion of p38γ and p38δ altered antigen-specific IgG subclass production *in vivo*, causing transient reductions in IgG2b and IgG3 and a persistent decrease in IgG2a titres. This defect correlated with impaired activation of CD4^+^ T cells, reduced generation of effector T cells, and a significant decrease in T follicular helper (Tfh) cell differentiation. Gene expression analyses revealed diminished levels of key Tfh-associated cytokines, including *Il21*, *Il4*, *Cxcl13*, and *Il10*. Together, these findings demonstrate that p38γ/p38δ signalling in T cells is essential for Tfh differentiation, cytokine production, and effective T cell-dependent humoral immunity, including the generation of class-switched IgG antibodies, particularly IgG2 subclasses.

## Introduction

The p38 MAPK family plays a central role in coordinating immune responses, yet the specific contribution of its members p38γ and p38δ (p38γ/p38δ) to adaptive immunity has only recently begun to be elucidated (1). While p38α has been extensively studied, emerging evidence indicates that p38γ/p38δ regulate distinct immunological processes, particularly within T cells. These kinases have been implicated in T cell activation, cytokine production, and the orchestration of inflammatory responses (1–4), raising the possibility that they may also influence the crosstalk between T and B cells that underlies humoral immunity.

T cells are essential for the activation of B cells in response to protein antigens (5). Previous work from our group has demonstrated that p38γ/p38δ are required for optimal production of antibodies against T-dependent antigens, as mice lacking both kinases exhibit reduced IgG titres in collagen-induced model (3), or following immunization with T-dependent antigen (6). However, because p38γ and p38δ are expressed in multiple immune cell populations, these studies did not establish whether the impaired antibody response resulted from T cell-intrinsic defects or from alterations in other cellular compartments.

T-dependent antibody responses rely on a coordinated sequence of events: antigen presentation by antigen-presenting cells (APCs), activation and differentiation of CD4^+^ T cells, formation of germinal centres, and the generation of high-affinity, class-switched antibodies (7–10). Follicular helper T (Tfh) cells are especially critical in this process, as they provide the signals required for B cell selection, survival, and class-switch recombination (11). However, the signalling pathways that govern Tfh differentiation and function remain incompletely understood.

Although our previous work showed that p38γ/p38δ-deficient mice have impaired antibody responses to T-dependent antigens, it remains unclear whether this defect reflects intrinsic alterations in T cells and whether p38γ and p38δ play redundant or distinct roles. Furthermore, the contribution of these kinases to T-cell differentiation and helper functions during humoral immune responses has not been directly addressed. To dissect the T cell-specific contribution of p38γ and p38δ to the humoral immune response, we employed an immunization model that induces a robust T-dependent response and allows precise monitoring of antibody production, T cell activation, Tfh differentiation, and germinal centre formation. Using conditional knockout mice lacking either p38γ, p38δ, or both kinases specifically in T cells, we investigated how these p38MAPKs shape the cellular and molecular events that drive antibody generation.

Our results reveal that both p38γ and p38δ together are essential regulators of T cell activation, Tfh differentiation, cytokine production, and ultimately, IgG class-switching. Together, these findings uncover a previously unappreciated role for these p38 MAPKs in adaptive immunity and provide new insight into the signalling pathways that control T cell-dependent humoral responses.

## Materials and methods

### Mice

C57BL/6J-CD4-Cre^+/--^p38γ/δ^f/f^ (CD4Cre-p38γ/δ^f/f^) mice were generated by crossing *Mapk12*/*Mapk13*^flox/flox^ (p38γ/δ^f/f^) (12) with B6.Cg-Tg(Cd4-cre)1Cwi/BfluJ (The Jackson Laboratory). To generate CD4Cre-p38γ^f/f^, CD4Cre-p38δ^f/f^ and their corresponding floxed control (p38γ^f/f^ and p38δ^f/f^), CD4Cre-p38γ/δ^f/f^ mice were crossed with C57BL/6J wild type animals and the resulting offspring intercrossed to obtain the desired genotypes. Experimental controls consisted of littermate floxed mice lacking the CD4-Cre transgene. Genotype were determined by PCR analysis of DNA isolated from tail biopsies using the primers listed in **Supplementary Table 1**. All genotypes were obtained at the expected Mendelian frequencies, exhibited normal fertility and viability, and showed no overt phenotypic abnormalities. Male and female mice aged 12–14 weeks were used for all experiments. Mice were housed in specific-pathogen-free conditions in accordance with European Union regulations; work was performed according to the regulation of local CNB and CSIC bioethics committees and approved by the Community of Madrid PROEX 316/15, 071/19 and 289.2/20.

### Immunization and determination of antigen-specific antibodies by ELISA

NP-KLH is a well-established model antigen for studying T-dependent humoral immune responses, and Alum is widely used as an adjuvant to enhance antigen-specific immunity (13). Mice were immunized intraperitoneally with NP-KLH (50 μg; Biosearch Technologies) in alum (100 μl; Thermo Scientific), in a final volume of 200 μl. Unless otherwise indicated, experiments were performed using groups of 4–5 mice per genotype at each time point. Peripheral blood samples were collected 1 day before immunization (day 0) and at days 7, 14, 21, and 28 post-immunization. Spleens were harvested at the indicated time points for cellular analyses.

Serum levels of NP-specific antibodies of different isotypes (IgM, IgG, IgG1, IgG2a, IgG2b, IgG3, and IgA) were determined by ELISA. Briefly, ELISA plates were coated with NP-BSA (ratio >20) (Biosearch Technologies), blocked with PBS containing 2% (w/v) BSA, and incubated with appropriately diluted serum samples (1:1000 for IgA and IgG subclasses, and 1:5000 for IgM). Bound antibodies were detected using biotin-conjugated goat anti-mouse isotype-specific antibodies (Southern Biotech) followed by alkaline phosphatase-streptavidin (Sigma). The colorimetric substrate p-nitrophenyl phosphate (Sigma) was added and absorbance was measured at 405 nm.

### T cell isolation

For T cell isolation, a total splenocyte suspension was obtained by dissociating the spleens from the animals through a 40 μm cell strainer. The suspension was centrifuged at 300 × *g* for 5 min at 4 °C, and erythrocytes were lysed for 5 min at room temperature. Cell purification was performed using the MojoSort™ Mouse CD4 T Cell Isolation Kit for CD4^+^ T cells or the MojoSort™ Mouse CD8 T Cell Isolation Kit for CD8^+^ T cells (BioLegend), following the manufacturer’s instructions. After isolation, the purity of the cell populations was assessed by flow cytometry, reaching at least 97.8% for CD4^+^ T cells and 96.0% for CD8^+^ T cells.

### Immunoblotting

Protein samples were obtained by lysing total tissue or isolated cells, with lysis buffer (50 mM Tris-HCl pH 7.5; 1 mM EGTA; 1 mM EDTA; 50 mM sodium fluoride; 10 mM sodium β-glycerophosphate; 5 mM pyrophosphate; 1 mM sodium orthovanadate; 0.27 M sucrose, 0.1 mM phenylmethylsulphonyl fluoride, 1% (v/v) Triton X-100, 1 mM benzamidine plus 0.1% (v/v) β-mercaptoethanol). Lysates were centrifuged in a bench top centrifuge (20800 x *g*, 15 min, 4°C) and supernatant were collected. Protein concentration was calculated by colorimetry using Bradford method. Supernatants were quick-frozen, and stored at -80 °C.

Protein samples (50 µg) were resolved in SDS-PAGE and transferred to nitrocellulose membranes, which were blocked (30 min) in 50 mM Tris/HCl (pH 7.5), 0.15 M NaCl, 0.05% (v/v) Tween (TBST buffer) containing 10% (w/v) non-fat dry milk, then incubated in TBST buffer with 5% (w/v) non-fat dry milk and 0.5-1 μg/ml antibody (2 h, room temperature or overnight, 4 °C). Protein was detected using AlexaFluor-conjugated secondary antibodies (Invitrogen), using the Odyssey infrared imaging system. Anti-p38γ and -p38δ antibodies were raised and purified as described (14). Anti-p38α and anti-tubulin antibodies were from Santa Cruz and Sigma-Aldrich, respectively. Immunoblot band intensities were quantified using Fiji (ImageJ, NIH). Integrated pixel densities were measured in manually defined rectangular regions of interest around each band, with local background subtraction. Signals were normalized to the corresponding loading control (tubulin or p38α) within each lane and expressed relative to the control genotype (set to 1). Densitometric values are shown below the representative blots.

### Flow cytometry analysis

Thymus, spleen and lymph node cell suspensions were prepared by mechanical disaggregation (6, 15). Flow cytometry analysis was performed with 1x10^6^ cells per condition resuspended in 3% (v/v) FBS-PBS; cells were stained with combinations of fluorescently labelled antibodies to the cell surface, indicated in the figures and in (**Supplementary Table 2**), and incubated at 4°C for 30 min. The analysis of cell populations was performed in a Gallios Analyzer or CYTOMICS FC 500 Analyzer (Beckman Coulter) using the software Kaluza V1.5 (Beckman Coulter) and FlowJo v10 (FlowJo).

### Gene expression analysis

For mRNA analysis, specific tissue or isolated cells were lysed with NZYol (NZYtech) and RNA extracted using a standard protocol with chloroform-isopropanol-ethanol. cDNA for real-time quantitative PCR (RT-qPCR) was generated from total RNA using the High Capacity cDNA Reverse Transcription Kit (Applied Biosystems) and as described (2). PCR reactions were carried out in a QuantStudio5 (Applied Biosystems) and SDS v2.2 software was used to analyse the results by the Comparative Ct Method (ΔΔCt). X-fold change in mRNA expression was quantified relative to the control *Actb* mRNA. The different genes analysed in this work were amplified using the primers described in (**Supplementary Table 3**).

### Restimulation of splenocytes with NP-KLH

To analyse cytokine production in cells isolated from the spleens of NP-KLH-immunized mice, 5 x 10^6^ splenocytes were cultured in 24-well plates in 1 mL of complete RPMI 1640 medium supplemented with 50 μg of NP-KLH antigen, added in soluble form to the culture medium, for 72 h. Cells were then lysed, and the mRNA levels of different cytokines were quantified by RT-qPCR.

### Statistical analyses

Statistical analyses were performed using GraphPad Prism v.7.0a. Antibody titre kinetics in serum samples from the same animals across multiple time points were analysed by repeated-measures two-way ANOVA, followed by Bonferroni’s multiple comparisons test. Comparisons between two groups only were performed using a two-tailed unpaired Student’s t-test. A *p*-value ≤ 0.05 was considered statistically significant in all cases.

## Results

### Generation of T cell-specific conditional p38γ and p38δ knockout mice

**Figure 1:**
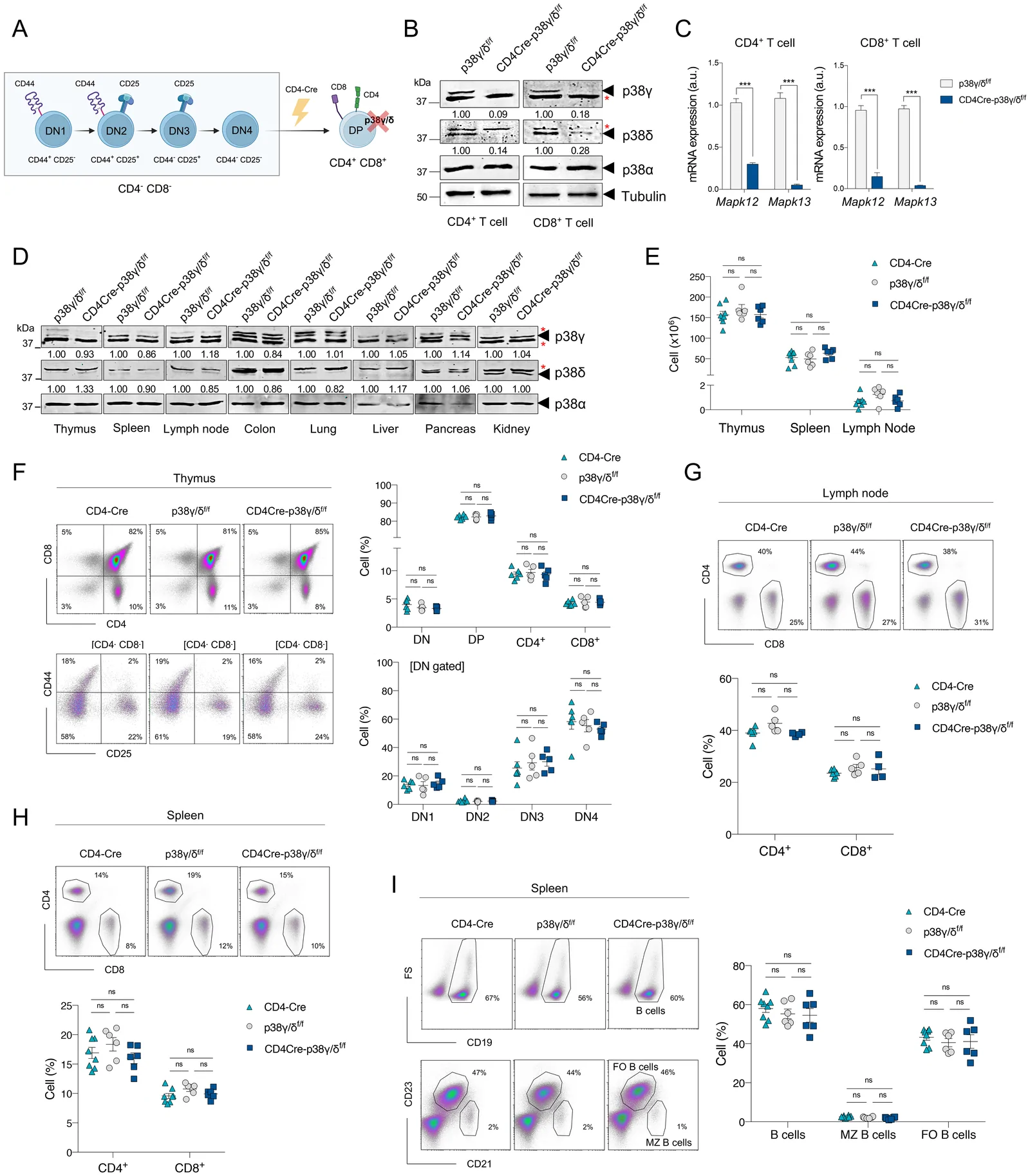
Characterization of the CD4Cre-p38γ/δ^f/f^ mouse line. **(A)** Schematic representation of the development of T cells from CD4^-^CD8^-^ double-negative (DN) to CD4^+^CD8^+^ double-positive (DP) in mouse thymus. DN T cell subpopulations are also shown: DN1 (CD25^-^CD44^+^), DN2 (CD25^+^CD44^+^), DN3 (CD25^+^CD44^-^), and DN4 (CD25^-^CD44^-^). CD4-driven Cre recombinase expression is initiated at the late DN/DP transition (indicated by the lightning bolt in the scheme), resulting in deletion of floxed alleles in developing CD4^+^ and CD8^+^ T cells. Consequently, mature T cells from CD4Cre-p38γ/δ^f/f^ mice lack p38γ and p38δ expression. **(B)** CD4^+^ or CD8^+^ T cell lysates (50 μg) from p38γ/δ^f/f^ and CD4Cre-p38γ/δ^f/f^ mice were immunoblotted with the specific antibodies to determine the expression of the indicated proteins. Representative blots are shown. Tubulin was used as loading control. Red asterisks indicate unspecific bands. **(C)** Relative mRNA expression of the indicated genes from CD4^+^ and CD8^+^ T cells was determined by qPCR. *Actb* was used as control. Results show means ± SEM (n = 8). a.u., arbitrary units. **(D)** Lysates (50 μg) of different tissues from p38γ/δ^f/f^ and CD4Cre-p38γ/δ^f/f^ mice were immunoblotted with the specific antibodies to determine the expression of the indicated proteins. p38α was used as loading control. Red asterisks indicate unspecific bands. **(E)** Number of cells in the thymus, spleen and lymph node of CD4-Cre, p38γ/δ^f/f^ and CD4Cre-p38γ/δ^f/f^ mice. Each dot represents a single mouse. (n = 6-8). **(F)** Cells from the thymus of CD4-Cre, p38γ/δ^f/f^ and CD4Cre-p38γ/δ^f/f^ mice were stained with anti-CD4, -CD8, -CD44 and -CD25 antibodies. The different populations represented (Double Negative (DN), Double Positive (DP), CD4^+^, CD8^+^, DN1, DN2, DN3 and DN4) were analysed by flow cytometry. Representative flow cytometry plots are shown. Percentage of the different T cell populations are shown as means ± SEM of two independent experiments. Each dot represents a single mouse. (n = 6-8). **(G)** Cells from the inguinal lymph nodes of CD4-Cre, p38γ/δ^f/f^ and CD4Cre-p38γ/δ^f/f^ mice were stained with anti-CD4 and -CD8 antibodies. The different populations represented (CD4^+^ and CD8^+^) were analysed by flow cytometry. Representative flow cytometry plots are shown. Percentage of the different T cell populations are shown as mean ± SEM of two independent experiments. Each dot represents a single mouse. (n = 6-8). **(H, I)** Cells from the spleen of CD4-Cre, p38γ/δ^f/f^ and CD4Cre-p38γ/δ^f/f^ mice were stained with anti-CD4, -CD8, -CD19, -CD21, and -CD23 antibodies. The different populations represented were analysed by flow cytometry. Representative flow cytometry plots are shown. FS, forward scatter. Percentage of the different T cell populations are shown as mean ± SEM of two independent experiments. Each dot represents a single mouse. (n = 6-8). In all panels, ns, non-significant; ***p ≤ 0.001.

We aimed to determine how p38γ/p38δ within T cells influence antibody production following immunization with T-dependent antigens. For this, we used mouse lines that specifically did not express these kinases in T cells, either in combination or individually (CD4Cre-p38γ/δ^f/f^, -p38γ^f/f^ and -p38δ^f/f^ mice). Because p38γ/p38δ are implicated in T cell development (15), we generated CD4Cre mouse lines in which Cre recombinase is expressed after CD4^-^ CD8^-^ double-negative thymocyte development (16) (**Figure 1A**). CD4Cre-p38γ/δ^f/f^ mice showed complete loss of the mRNA and protein expression of both p38γ/p38δ (*Mapk12*/*Mapk13*) in CD4^+^ and CD8^+^ T cells (**Figures 1B, C**), whereas their expression in other tissues remained unchanged (**Figure 1D**). Likewise, T cells from CD4Cre-p38γ^f/f^ mice lacked p38γ and *Mapk12* expression (**Supplementary Figures 1A, B**), and T cells from CD4Cre-p38δ^f/f^ mice lacked p38δ and *Mapk13* expression (**Supplementary Figures 1D, E**), without alterations in their levels in other tissues (**Supplementary Figures 1C, F**).

Importantly, the total number of thymocytes, splenocytes and peripheral lymph node cells (**Figure 1E**), as well as the distribution of major T cell subsets in thymus, lymph nodes, and spleen, were comparable across CD4Cre-p38γ/δ^f/f^, p38γ/δ^f/f^ or CD4-Cre control mice (**Figures 1F–H**). As expected from the timing of CD4-Cre expression, no major alterations in thymocyte differentiation were observed in CD4Cre-p38γ/δ^f/f^ mice (**Figure 1F**). We also analysed B cells, as well as the conventional mature B cell subset, follicular (FO) and marginal zone (MZ) B cells in the spleen (**Figure 1I**). All total, FO and MZ B cell frequency were not affected by the lack of p38γ/p38δ in T cells (**Figure 1I**). These data indicate that deletion of p38γ and p38δ in T cells does not impair thymic development or the establishment of normal peripheral T cell populations, ensuring that any subsequent immunological differences arise from functional rather than developmental defects.

### p38γ and p38δ are needed for optimal T cell-dependent humoral immune response

**Figure 2:**
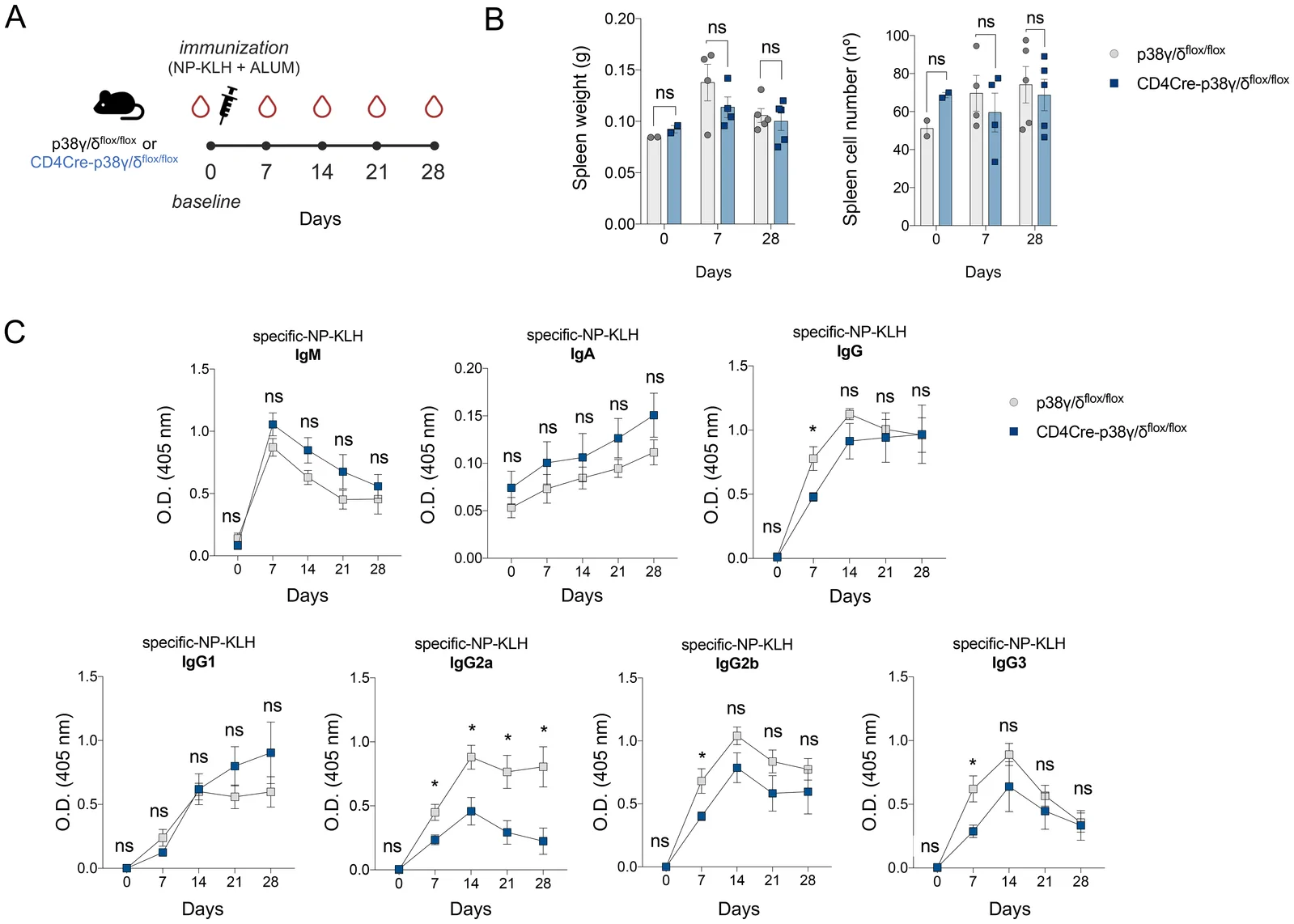
Antibody response to T-dependent antigen in p38γ/δ^f/f^ and CD4Cre-p38γ/δ^f/f^ mice. **(A)** Schematic representation of the T cell-dependent antigen immunization model. p38γ/δ^f/f^ and CD4Cre-p38γ/δ^f/f^ mice were injected intraperitoneally with 50 μg of NP-KLH using Alum as an adjuvant. Blood samples were collected weekly for serum antibody titter determination on days 0 (pre-immunization), 7, 14, 21 and 28 post-immunization. **(B)** Spleen weight and total splenocyte number in mice at days 0, 7, and 28 post-immunization. Each symbol represents a single mouse (n=2-5). **(C)** Serum titers of NP-KLH–specific antibodies in p38γ/δ^f/f^ and CD4Cre-p38γ/δ^f/f^ mice, determined by ELISA. Each symbol represents the mean ± SEM of 4–5 mice per group. In all panels, ns = non-significant; *p ≤ 0.05.

To determine the physiological relevance of p38γ and p38δ in T cell-dependent humoral responses, we immunized p38γ/δ^f/f^ and CD4Cre-p38γ/δ^f/f^ mice with NP-KLH and monitored the immune response over time (**Figure 2A**). Spleen size and total splenocyte numbers were comparable between genotypes at all time points (**Figure 2B**). Similar results were obtained in CD4Cre-p38γ^f/f^ and CD4Cre-p38δ^f/f^ mice and their respective controls (**Supplementary Figures 2A, B**).

In parallel, to investigate the *in vivo* function of p38γ/p38δ in T cells in the antibody production during the humoral response, we measured by ELISA the levels of different classes of NP-specific antibodies (IgM, IgG, IgG1, IgG2a, IgG2b, IgG3, and IgA) in the serum of p38γ/δ^f/f^ and CD4Cre-p38γ/δ^f/f^ mice pre- and post-immunization (**Figure 2C**). Analysis revealed a rapid increase in IgM production in both genotypes, peaking during the first week after immunization and then declining quickly, while there was a mild induction of IgA over time (**Figure 2C**). We found that CD4Cre-p38γ/δ^f/f^ mice had significantly lower IgG antibody response than p38γ/δ^f/f^ mice. In particular, these mice showed an overall reduction of approximately 60% in IgG2a and around 30% in IgG2b and IgG3–7 days after immunization, whereas IgG1 production was similar in both genotypes (**Figure 2C**). Analysis of the antibody production in the single-knockout mice showed no major differences between CD4Cre-p38γ^f/f^ mice and their controls (**Supplementary Figure 2C**). However, CD4Cre-p38δ^f/f^ mice showed a significant, although modest, reduction in IgG2a levels compared with p38δ^f/f^ mice, together with a transient decrease in IgG2b levels at day 7 (**Supplementary Figure 2D**). These alterations were less pronounced that those observed in CD4Cre-p38γ/δ^f/f^ mice. These results show that maximal impairment of antibody production requires the combined absence of both p38γ and p38δ kinases, and indicate that p38γ/p38δ in T cells participate in the T-dependent humoral response by positively regulating IgG2 antibody production.

### p38γ/p38δ deficiency impairs the activation of CD4^+^ T cells and effector differentiation

**Figure 3:**
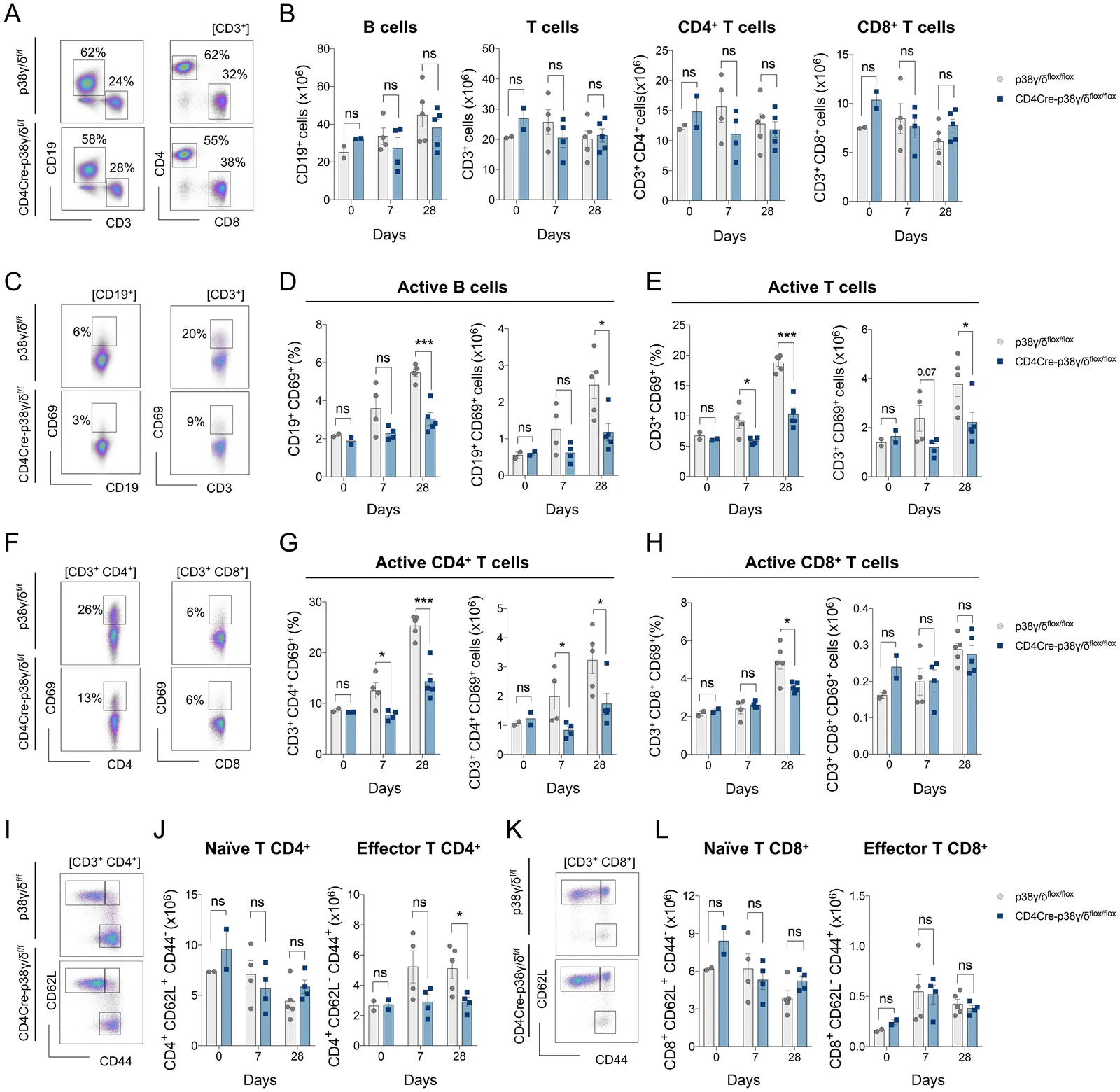
Analysis of splenic lymphocyte populations in p38γ/δ^f/f^ and CD4Cre-p38γ/δ^f/f^ mice following NP-KLH/Alum immunization. p38γ/δ^f/f^ and CD4Cre-p38γ/δ^f/f^ mice were immunized with NP-KLH + Alum and spleens were harvested at 0, 7, and 28 days post-immunization. **(A)** Flow cytometry gating strategy for the analysis of B and T cell populations in the spleens of p38γ/δ^f/f^ and CD4Cre-p38γ/δ^f/f^ mice at day 28 following NP-KLH/Alum immunization. **(B)** Number of splenic B cells and T cells at different time points of the immunization process. **(C-E)** Flow cytometry gating strategy for the analysis of CD69 expression on splenic B and T cells from of p38γ/δ^f/f^ and CD4Cre-p38γ/δ^f/f^ mice at day 28 following NP-KLH/Alum immunization. Percentage and number of activated **(D)** B cells and activated **(E)** T cells in the spleen at different time points. **(F-H)** Flow cytometry gating strategy for the analysis of CD69 expression on splenic CD4^+^ and CD8^+^ T cells from of p38γ/δ^f/f^ and CD4Cre-p38γ/δ^f/f^ mice at day 28 following NP-KLH/Alum immunization. Percentage and number of activated **(G)** CD4^+^ T cells and activated **(H)** CD8^+^ T cells in the spleen. **(I, K)** Flow cytometry gating strategy for the analysis of **(I)** CD4^+^ and **(K)** CD8^+^ naïve and effector T cell populations in the spleens of p38γ/δ^f/f^ and CD4Cre-p38γ/δ^f/f^ mice at day 28 following NP-KLH/Alum immunization. **(J, L)** Numbers of naïve (CD4^+^CD62L^+^CD44^-^) and effector (CD4^+^CD62L^-^CD44^+^) **(J)** CD4^+^ T cells and **(L)** CD8^+^ T cells in the spleen. In all panels, data are shown as mean ± SEM. Each dot represents a single mouse. (n=2-5). ns = non-significant; *p ≤ 0.05, ***p ≤ 0.001.

Following immunization, APCs capture and process the antigen and present it to T and B cells, leading to their activation, proliferation and differentiation (17). To determine whether these processes were altered in CD4Cre-p38γ/δ^f/f^ mice, we analysed splenic T- and B-cell populations and their activation status throughout the immunization protocol. No differences were observed between genotypes in the total number of B cells, T cells, or CD4^+^ and CD8^+^ T cell populations (**Figures 3A, B**). However, analysis of the activation marker CD69 revealed a reduction in the number of activated B and T cells in the spleens of CD4Cre-p38γ/δ^f/f^ mice compared with p38γ/δ^f/f^ controls (**Figures 3C–E**). The decrease in T cells was specifically attributable to reduced activation of CD4^+^ T cells (**Figures 3F, G**). Activated CD8^+^ T cells showed only a modest increase during immunization and did not significantly differ between genotypes (**Figures 3F, H**). Importantly, analysis of mice lacking either p38γ or p38δ alone did not reveal significant differences in T or B cell total numbers or in their activation status compared with their corresponding control mice (**Supplementary Figures 3A–D**). These findings suggest that p38γ and p38δ exert redundant or cooperative functions during CD4^+^ T cell activation following immunization.

To further characterize T cell responses, we analysed the expression of CD44 and CD62L, markers commonly used to assess T cell activation and differentiation following immunization. Changes in the expression of these molecules are associated with the generation of effector and memory T cell subsets that support antigen-specific antibody responses (18). We found that the number of effector CD4^+^ T cells increased progressively in p38γ/δ^f/f^ mice during immunization, whereas this expansion was significantly impaired in CD4Cre-p38γ/δ^f/f^ mice (**Figures 3I, J**). By contrast, the proportions of naïve CD4^+^ T cell populations were comparable between genotypes (**Figures 3I, J**). No significant alterations were detected in CD8^+^ T cells (**Figures 3K, L**), indicating that the differences observed within the total T cell compartment were specifically attributable to a defect in CD4^+^ T cell generation.

Together, these results indicate that p38γ/p38δ signalling is required for efficient activation and acquisition of the effector phenotype by CD4^+^ T cells in secondary lymphoid organs following immunization.

### Tfh differentiation and cytokine production in T-dependent immunization are controlled by p38γ/p38δ

**Figure 4:**
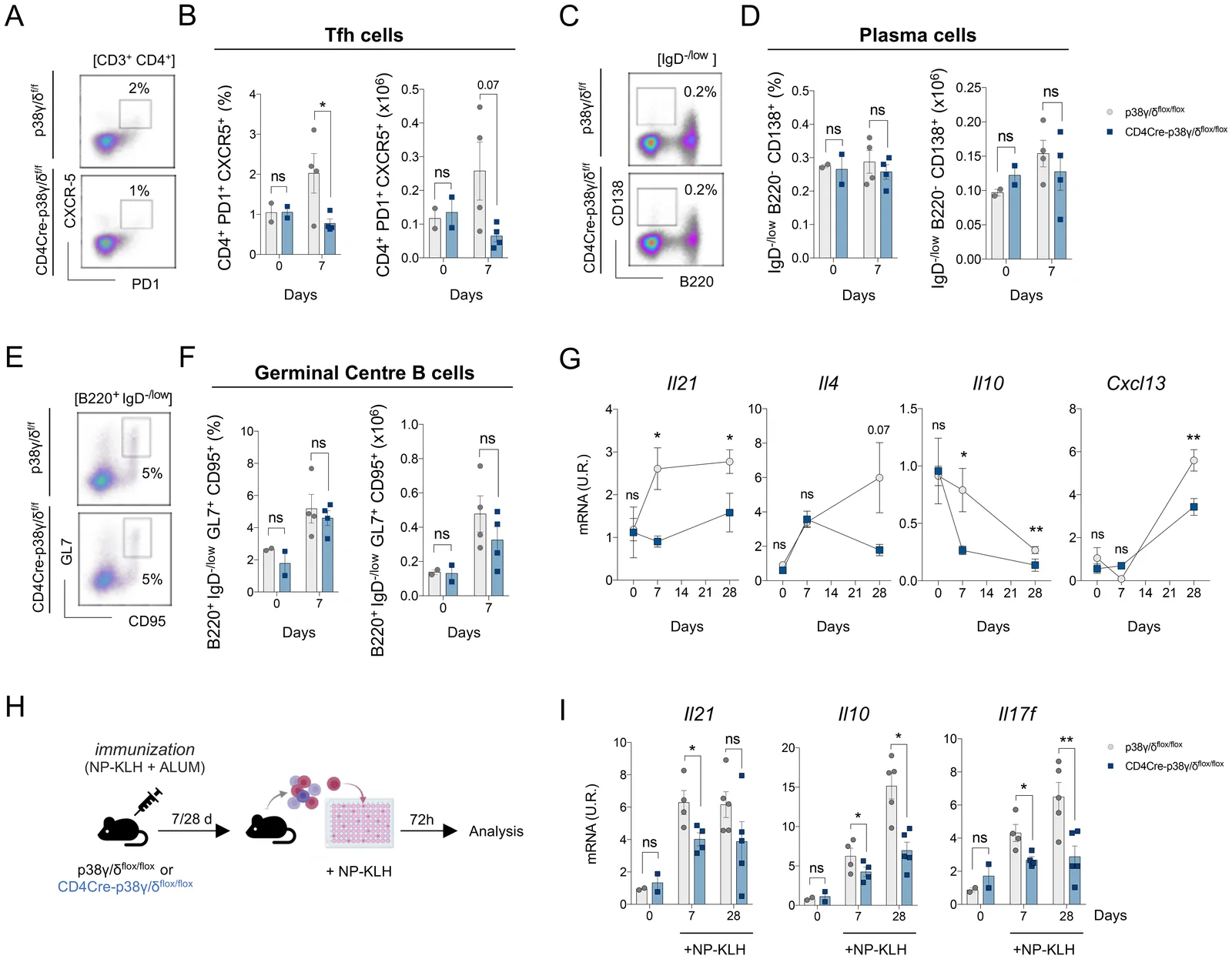
Impaired Tfh differentiation and cytokine production following immunization in CD4Cre-p38γ/δ^f/f^ mice. p38γ/δ^f/f^ and CD4Cre-p38γ/δ^f/f^ mice were immunized with NP-KLH + Alum and spleens were harvested at different times post-immunization. Day 0 corresponds to non-immunized mice, whereas days 7, 14 and 28 indicate the indicated times after NP-KLH immunization. **(A, C, E)** Flow cytometry gating strategy for the identification of T follicular helper (Tfh) cells (CD4^+^PD1^+^CXCR5^+^), plasma cells (IgD^-/low^B220^-^CD138^+^), and germinal centre B cells (B220^+^IgD^-/low^GL7^+^CD95^+^), respectively, in the spleens of p38γ/δ^f/f^ and CD4Cre-p38γ/δ^f/f^ mice at day 7 following NP-KLH/Alum immunization. **(B, D, F)** Percentage and number of **(B)** Tfh cells, **(D)** plasma cells, and **(F)** germinal centre B cells in the spleen of p38γ/δ^f/f^ and CD4Cre-p38γ/δ^f/f^ mice. Data are shown as mean ± SEM. Each dot represents a single mouse. (n = 2-4). **(G)** RT-qPCR analysis of cytokine gene expression in spleens from p38γ/δ^f/f^ and CD4Cre-p38γ/δ^f/f^ mice following NP-KLH/Alum immunization. *Actb* was used as control. Each symbol represents the mean ± SEM of 2–5 mice per group. **(H)** Schematic representation of the immunization protocol and the re-stimulation with NP-KLH *in vitro* of splenocytes during 72h. **(I)** RT-qPCR analysis of cytokine gene expression in splenocytes isolated from p38γ/δ^f/f^ and CD4Cre-p38γ/δ^f/f^ mice and re-stimulated *in vitro* with 50 μg NP-KLH for 72 h. *Actb* was used as control. Each symbol represents a single mouse. (n=2-5). In all panels, ns, non-significant; *p ≤ 0.05, **p ≤ 0.01.

Given the impaired activation and effector differentiation of CD4^+^ T cells observed in CD4Cre-p38γ/δ^f/f^ mice, we next investigated whether the generation of cellular populations directly involved in humoral immune responses was also affected. In particular, we analysed Tfh cells, germinal centre (GC) B cells, and plasma cells, as these populations are essential for GC formation, affinity maturation, class-switch recombination, and the production of high-affinity antibodies following immunization (11). During the first days after immunization, activated CD4^+^ T cells differentiate into Tfh cells, a process characterized by the upregulation of CXCR5 and PD-1 expression (5). While p38γ/δ^f/f^ mice showed the expected increase in Tfh cell numbers at day 7 post-immunization, this expansion was markedly impaired in CD4Cre-p38γ/δ^f/f^ mice (**Figures 4A, B**). No significant differences in Tfh cell frequencies were detected between genotypes in non-immunized mice (day 0). These results suggest that p38γ/p38δ signalling contributes to the differentiation and/or maintenance of Tfh cells during the humoral response. Importantly, mice lacking either p38γ or p38δ alone did not show significant differences in Tfh cell numbers compared with their corresponding control mice (**Supplementary Figures 4A, B**), supporting redundant or cooperative functions of these kinases in T cells.

We next analysed GC B cells and plasma cells in the spleens of p38γ/δ^f/f^ and CD4Cre-p38γ/δ^f/f^ mice at days 0 and 7 post-immunization, when mature GCs are established (5). No significant differences were observed between genotypes in either population (**Figures 4C–F**). Similarly, mice lacking either p38γ or p38δ alone showed no alterations compared with their respective controls (**Supplementary Figures 4C–F**).

T cell-derived cytokines and chemokines are essential for effective T-dependent humoral responses. Molecules such as IL-21, IL-4, IL-10, and CXCL13 are required for proper GC formation and maintenance, as well as for optimal B cell activation and differentiation (19, 20). We assessed whether p38γ/p38δ deficiency affected their expression, and found that in the spleen of CD4Cre-p38γ/δ^f/f^ mice, *Il21*, *Il4*, *Il10* and *Cxcl13* mRNA levels were lower than in that of p38γ/δ^f/f^ mice following immunization (**Figure 4G**).

To further evaluate antigen-specific T cell responses, splenocytes obtained from p38γ/δ^f/f^ and CD4Cre-p38γ/δ^f/f^ mice, at different times after immunization, were restimulated *in vitro* with NP-KLH for 72 h (**Figure 4H**). RT-qPCR analysis revealed reduced expression of *Il2*, *Il10* and *Il17f* mRNA in splenocytes from CD4Cre-p38γ/δ^f/f^ mice compared with controls (**Figure 4I**).

Taken together, these results indicate that p38γ/p38δ signalling in T cells is required for the proper development of T-dependent humoral responses. Combined deletion of p38γ and p38δ impairs Tfh cell generation, reduces CD4^+^ T cell activation and effector differentiation, and alters the production of cytokines involved in GC responses following immunization. These defects likely contribute to the reduced antibody titres observed in CD4Cre-p38γ/δ^f/f^ mice compared with p38γ/δ^f/f^ controls.

## Discussion

Using the NP-KLH immunization model, we investigated the contribution of p38γ and p38δ to T-dependent antibody responses. Our results showed that p38γ and p38δ expression in the T cell compartment cooperate to regulate specific class-switched antibody production *in vivo*. Although CD4^+^ T cell-specific deletion of p38δ alone resulted in a reduction in IgG2a and IgG2b titres, this effect was only partial. In contrast, the simultaneous loss of both kinases produced a more pronounced impairment, indicating that T cell-intrinsic p38γ and p38δ exert overlapping or complementary functions in the control of T cell-dependent antibody responses, consistent with the possibility that p38γ and p38δ have partially redundant functions. These findings are consistent with previous studies showing impaired IgG2 responses in mice globally deficient for both p38γ and p38δ following NP-KLH immunization, whereas B cell-specific deletion of these kinases did not affect antibody production (6). Together, these observations suggest that CD4^+^ T cells represent an important cellular compartment through which p38γ and p38δ regulate antibody responses, whereas B cell-intrinsic expression of these kinases appears dispensable in this model. Furthermore, our previous work in the collagen-induced arthritis model showed that p38γ/δ-deficient mice exhibit reduced IgG2 levels following collagen challenge, supporting the involvement of these kinases in class-switched antibody responses across different experimental settings (3). The reduction in IgG2 subclasses may have important functional consequences beyond humoral immunity. IgG2a, IgG2b, and IgG3 display high affinity for activating Fcγ receptors and are therefore particularly effective in mediating effector mechanisms such as antibody-dependent cellular cytotoxicity and inflammatory responses (21).

Mechanistically, the impaired antibody response observed in p38γ/δ-deficient mice was associated with alterations in the CD4^+^ T cell compartment. Following immunization, CD4Cre-p38γ/δ^f/f^ mice exhibited reduced numbers of effector CD4^+^ T cells and Tfh cells, two populations that are essential for the development of productive germinal centre reactions and high-affinity antibody responses (7, 9, 11). Interestingly, despite the reduction in Tfh cells, the frequencies of germinal centre B cells and plasma cells were not significantly altered. These observations suggest that p38γ/p38δ signalling may primarily affect the functional quality of T cell help rather than the overall generation of B cell effector populations. Such a scenario could explain why antibody production is impaired even in the absence of major quantitative defects in downstream B cell compartments.

Although activated CD8^+^ T cells also showed genotype-dependent differences, these were considerably less pronounced than those observed in CD4^+^ T cells. Thus, while a broader role for p38γ/δ in T-cell activation cannot be excluded, their functional impact in this model appears most evident in the CD4^+^ T-cell compartment.

In line with this interpretation, we observed decreased expression of IL-4 and IL-21 in the spleens of immunized CD4Cre-p38γ/δ^f/f^ mice. Both cytokines play central roles in humoral immunity, but IL-21 is particularly important for germinal centre maintenance, plasma cell differentiation, and immunoglobulin class switching (22). Together with CD40 signalling, IL-21 promotes the differentiation of naïve B cells into plasma cells and drives immunoglobulin class switching from IgM to IgG (22). Interestingly, *ex vivo* restimulation of splenocytes from immunized mice with NP-KLH antigen reproduced several of the cytokine defects observed *in vivo*, including a significant reduction in IL-17F, IL-10 and IL-21 production in CD4Cre-p38γ/δ^f/f^ mice. These findings suggest that the impaired cytokine response is not exclusively dependent on environmental cues present during the immune response *in vivo*, but rather is consistent with an intrinsic requirement for p38γ/p38δ signalling in the generation or maintenance of functional Tfh-like responses. Given the well-established role of IL-21 as a key mediator of germinal centre reactions and antibody class switching, defective IL-21 production by T cells may contribute to the diminished IgG2 responses observed in p38γ/δ-deficient mice.

An important aspect of our findings concerns the temporal relationship between the humoral and cellular phenotypes. Reduced antibody titers were most evident during the early phase of the response, coinciding with a reduction in Tfh cells at the same time point, while effector CD4^+^ T cells showed a similar downward trend that became more pronounced at later time points. Although the current data do not establish a direct causal relationship between these observations, they suggest that p38γ/p38δ signalling may influence T cell activation and help from the early stages of the response. The concurrent reduction in Tfh cells and antibody titers, together with the reduced IL-21 production observed in CD4Cre-p38γ/δf/f mice, suggests that early alterations in T cell helper function may contribute to impaired antibody production. The progressive reduction in effector CD4^+^ T cells may reflect a defect in the maintenance or persistence of this population. Further kinetic analyses will be required to determine the precise temporal window during which p38γ/δ signalling influences T cell help and antibody responses.

While our data demonstrate an important role for p38γ/p38δ in CD4^+^ T cell-mediated humoral immunity, the molecular mechanisms underlying this phenotype remain to be fully characterized. Future studies should address how p38γ/p38δ signalling controls the transcriptional and functional programs associated with Tfh differentiation and cytokine production. Moreover, given the key contribution of conventional type 2 dendritic cells (cDC) to antigen presentation, CD4^+^ T cell activation and polarization, and cytokine production following immunization (23), it will be of interest to determine whether p38γ/p38δ signalling in cDC cooperates with T cell-intrinsic pathways to optimize antibody responses.

In summary, this study identifies p38γ and p38δ expression in the CD4 T cell compartment as important regulators of T cell-dependent antibody response. By controlling CD4^+^ T cell activation, Tfh cell differentiation and cytokine production, these kinases promote effective antibody responses to T-dependent antigens. These findings reveal an important role for alternative p38 MAPK signalling in the coordination of adaptive immune responses and provide new insights into the mechanisms that link T cell function to antibody production.

## Data Availability

The original contributions presented in the study are included in the article/**Supplementary Material**. Further inquiries can be directed to the corresponding authors.
