# Supplementary material for: p38γ and p38δ support CD4+ T cell responses and T cell-dependent humoral immunity
Source: Front Immunol. 2026 Aug 26;17:1927358. doi: 10.3389/fimmu.2026.1927358 (PMC13553806; doi:10.3389/fimmu.2026.1927358)
Supplement: Supplementary file 1 [file DataSheet1.pdf]

Supplementary Figure legends

Supplementary Figure 1. **Characterization of the CD4Cre-p38γ$^{f/f}$ and CD4Cre-p38δ$^{f/f}$ mice.**

(A, D) 50 μg of lysate from CD4^{+} or CD8^{+} T cell purified from the spleen of (A) CD4Cre-p38γ$^{f/f}$ and p38γ$^{f/f}$ mice or (D) CD4Cre-p38δ$^{f/f}$ and p38δ$^{f/f}$ mice were immunoblotted with the specific antibodies to determine the expression of the indicated proteins. Representative blots are shown. Tubulin was used as loading control. Red asterisks indicate unspecific bands. **(B, E)** Relative mRNA expression of the indicated genes from splenic isolated CD4^{+} and CD8^{+} T cells from (B) CD4Cre-p38γ$^{f/f}$ and p38γ$^{f/f}$ mice or (E) CD4Cre-p38δ$^{f/f}$ and p38δ$^{f/f}$ mice, was determined by qPCR. *Actb* was used as control. Results show means ± SEM (n = 2-4). **(C, F)** Lysates (50 μg) of different tissues from (C) CD4Cre-p38γ$^{f/f}$ and p38γ$^{f/f}$ mice or (F) CD4Cre-p38δ$^{f/f}$ and p38δ$^{f/f}$ mice were immunoblotted with the specific antibodies to determine the expression of the indicated proteins. Representative blots are shown. p38α was used as loading control. L. Node, lymph node. Red asterisks indicate unspecific bands. In all panels, ns, non-significant; $^{**}p \leq 0.01$; $^{***}p \leq 0.001$.

Supplementary Figure 2. Antibody response to T-dependent antigen in CD4Cre-p38γ$^{f/f}$ and CD4Cre-p38δ$^{f/f}$ mice.

(A-B) Spleen weight and total splenocyte number of (A) p38γ$^{f/f}$ and CD4Cre-p38γ$^{f/f}$ mice, and (B) p38δ$^{f/f}$ and CD4Cre-p38δ$^{f/f}$ mice at days 0, 7, and 28 post-immunization. **(C-D)** Serum titers of NP-KLH–specific antibodies in (C) p38γ$^{f/f}$ and CD4Cre-p38γ$^{f/f}$, and (D) p38δ$^{f/f}$ and CD4Cre-p38δ$^{f/f}$ mice, determined by ELISA. Each symbol represents the mean ± SEM of 2-7 mice per group. ns = non-significant; $^{*}p \leq 0.05$, $^{**}p \leq 0.01$.

Supplementary Figure 3. Analysis of splenic lymphocyte populations in CD4Cre-p38γ$^{f/f}$ and CD4Cre-p38δ$^{f/f}$ mice following NP-KLH/Alum immunization.

p38γ$^{f/f}$, CD4Cre-p38γ$^{f/f}$, p38δ$^{f/f}$ and CD4Cre-p38δ$^{f/f}$ mice were immunized with NP-KLH + Alum and spleens were harvested at 0, 7, and 28 days post-immunization. **(A-B)** Number of splenic B cells and T cells at different time-points from (A) p38γ$^{f/f}$ and CD4Cre-p38γ$^{f/f}$ mice, and (B) p38δ$^{f/f}$ and CD4Cre-p38δ$^{f/f}$ mice. **(C-D)** Number of activated B cells and activated T cells in the spleen of (C) p38γ$^{f/f}$ and CD4Cre-p38γ$^{f/f}$ mice, and (D) p38δ$^{f/f}$ and CD4Cre-p38δ$^{f/f}$ mice. In all panels, data are shown as mean ± SEM. Each dot represents a single mouse. (n=2-7). ns = non-significant.

Supplementary Figure 4. Tfh differentiation and cytokine production following immunization in CD4Cre-p38γ$^{f/f}$ and CD4Cre-p38δ$^{f/f}$ mice.

(A, C, E) Left, flow cytometry gating strategy for the identification of T follicular helper (Tfh) cells ($CD4^+PD1^+CXCR5^+$), plasma cells ($IgD^{-/low}B220^-CD138^+$), and germinal centre B cells ($B220^+IgD^{-/low}GL7^+CD95^+$), respectively, in the spleens of p38γ$^{f/f}$ and CD4Cre-p38γ$^{f/f}$ mice at day 0 and 7 following NP-KLH/Alum immunization. Right, number and percentage of (A) Tfh cells, (C) plasma cells, and (E) germinal centre B cells in the spleen of p38γ$^{f/f}$ and CD4Cre-p38γ$^{f/f}$ mice. Data are shown as mean ± SEM. **(B, D, F)** Left, flow cytometry gating strategy for the identification of T follicular helper (Tfh) cells ($CD4^+PD1^+CXCR5^+$), plasma cells ($IgD^{-/low}B220^-CD138^+$), and germinal centre B cells ($B220^+IgD^{-/low}GL7^+CD95^+$), respectively, in the spleens of p38δ$^{f/f}$ and CD4Cre-p38δ$^{f/f}$ mice at day 0 and 7 following NP-KLH/Alum immunization. Right, number and percentage of (B) Tfh cells, (D) plasma cells, and (F) germinal centre B cells in the spleen of p38δ$^{f/f}$ and CD4Cre-p38δ$^{f/f}$ mice. In all panels, data are shown as mean ± SEM. Each symbol represents a single mouse. (n=2-7). ns = non-significant.

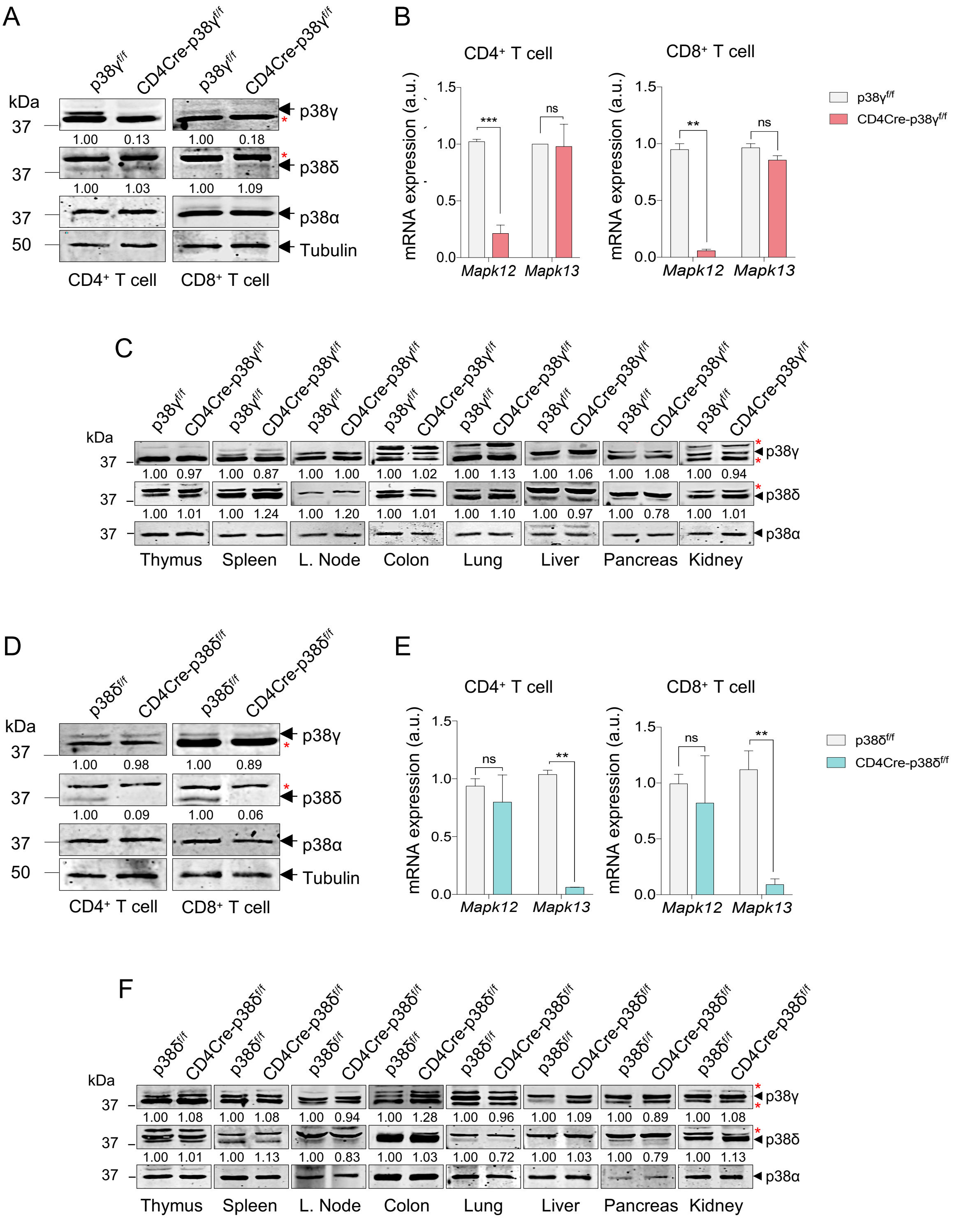
A
p38γf/f
CD4Cre-p38γf/f
p38γf/f
CD4Cre-p38γf/f
kDa
37
1.00 0.13
1.00 0.18
p38γ
37
1.00 1.03
1.00 1.09
p38δ
37
p38α
50
Tubulin
CD4+ T cell
CD8+ T cell
B
CD4+ T cell
CD8+ T cell
mRNA expression (a.u.)
1.5
1.0
0.5
0.0
***
ns
**
ns
Mapk12
Mapk13
p38γf/f
CD4Cre-p38γf/f
C
kDa
37
1.00 0.97 1.00 0.87 1.00 1.00 1.00 1.02 1.00 1.13 1.00 1.06 1.00 1.08 1.00 0.94
p38γ
37
1.00 1.01 1.00 1.24 1.00 1.20 1.00 1.01 1.00 1.10 1.00 0.97 1.00 0.78 1.00 1.01
p38δ
37
p38α
Thymus
Spleen
L. Node
Colon
Lung
Liver
Pancreas
Kidney
D
p38δf/f
CD4Cre-p38δf/f
p38δf/f
CD4Cre-p38δf/f
kDa
37
1.00 0.98
1.00 0.89
p38γ
37
1.00 0.09
1.00 0.06
p38δ
37
p38α
50
Tubulin
CD4+ T cell
CD8+ T cell
E
CD4+ T cell
CD8+ T cell
mRNA expression (a.u.)
1.5
1.0
0.5
0.0
ns
**
ns
**
Mapk12
Mapk13
p38δf/f
CD4Cre-p38δf/f
F
kDa
37
1.00 1.08 1.00 1.08 1.00 0.94 1.00 1.28 1.00 0.96 1.00 1.09 1.00 0.89 1.00 1.08
p38γ
37
1.00 1.01 1.00 1.13 1.00 0.83 1.00 1.03 1.00 0.72 1.00 1.03 1.00 0.79 1.00 1.13
p38δ
37
p38α
Thymus
Spleen
L. Node
Colon
Lung
Liver
Pancreas
Kidney

Suppl. Fig. 1

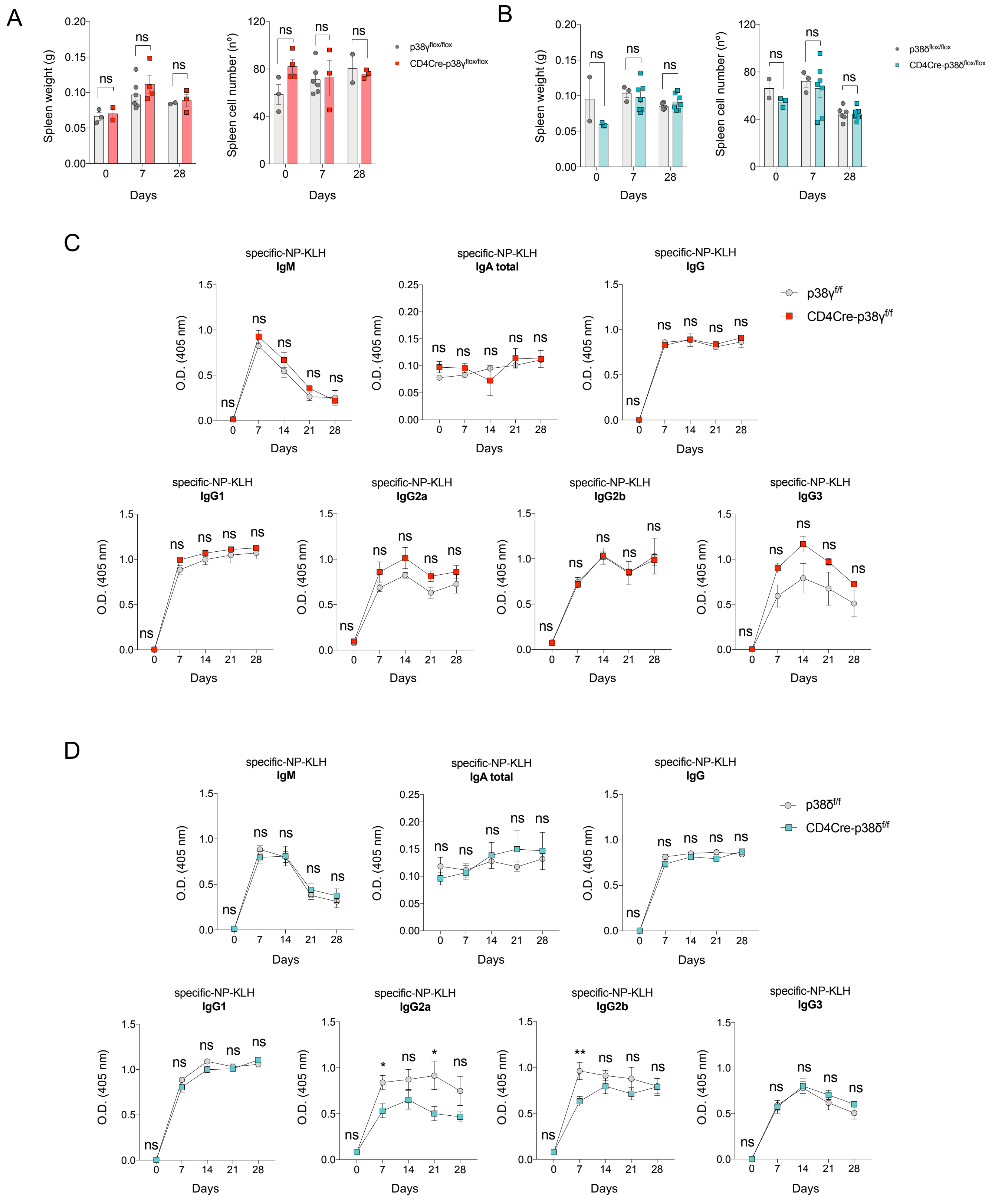
A
Spleen weight (g)
Spleen cell number (nº)
Days
ns
p38γ^flox/flox
CD4Cre-p38γ^flox/flox
B
Spleen weight (g)
Spleen cell number (nº)
Days
ns
p38δ^flox/flox
CD4Cre-p38δ^flox/flox
C
specific-NP-KLH
IgM
IgA total
IgG
IgG1
IgG2a
IgG2b
IgG3
O.D. (405 nm)
Days
ns
p38γ^f/f
CD4Cre-p38γ^f/f
D
specific-NP-KLH
IgM
IgA total
IgG
IgG1
IgG2a
IgG2b
IgG3
O.D. (405 nm)
Days
ns
*
**
p38δ^f/f
CD4Cre-p38δ^f/f

Suppl. Fig. 2

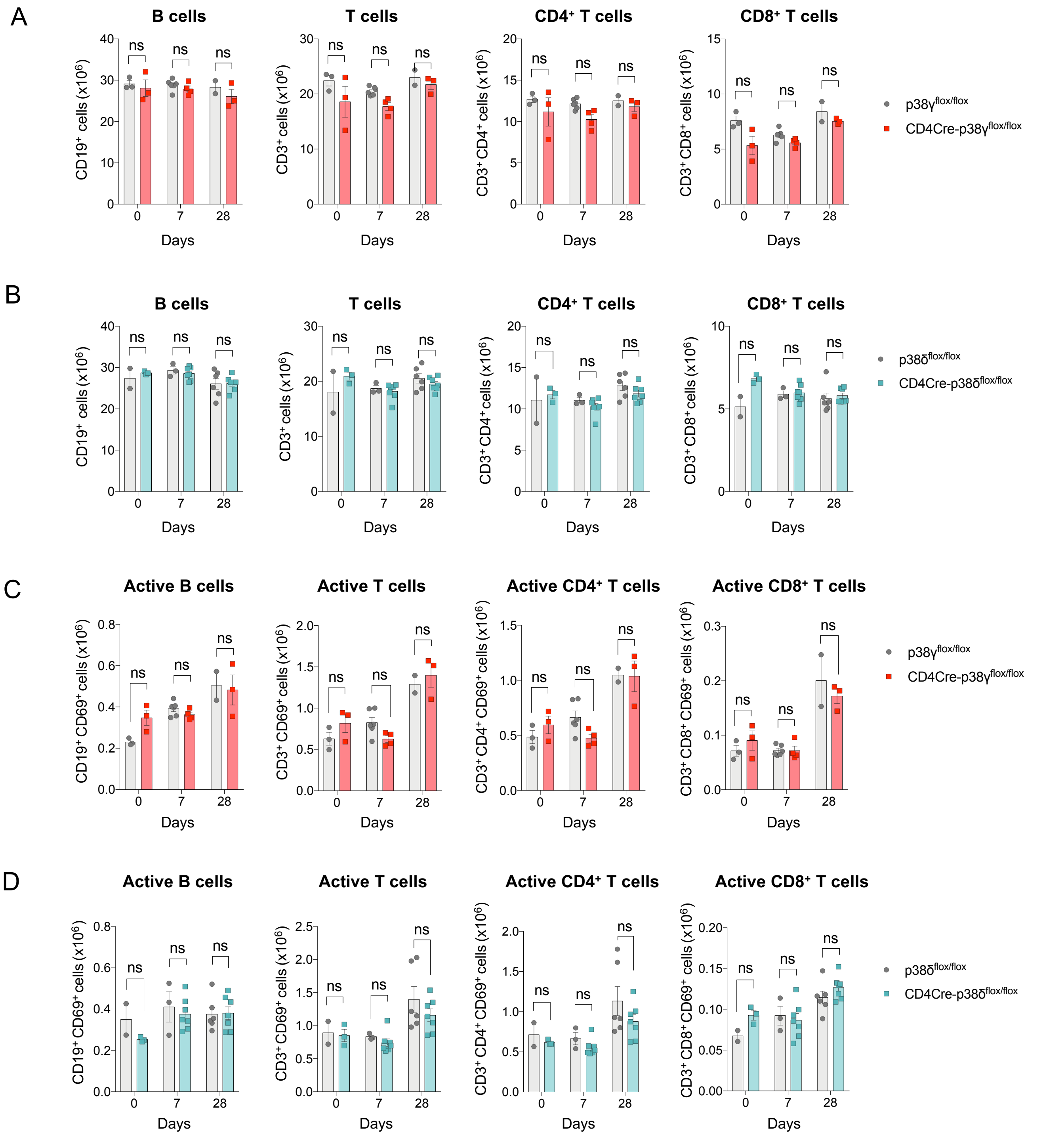
A
B cells
T cells
CD4+ T cells
CD8+ T cells
CD19+ cells (x10^6)
CD3+ cells (x10^6)
CD3+ CD4+ cells (x10^6)
CD3+ CD8+ cells (x10^6)
p38γ flox/flox
CD4Cre-p38γ flox/flox
B
B cells
T cells
CD4+ T cells
CD8+ T cells
CD19+ cells (x10^6)
CD3+ cells (x10^6)
CD3+ CD4+ cells (x10^6)
CD3+ CD8+ cells (x10^6)
p38δ flox/flox
CD4Cre-p38δ flox/flox
C
Active B cells
Active T cells
Active CD4+ T cells
Active CD8+ T cells
CD19+ CD69+ cells (x10^6)
CD3+ CD69+ cells (x10^6)
CD3+ CD4+ CD69+ cells (x10^6)
CD3+ CD8+ CD69+ cells (x10^6)
p38γ flox/flox
CD4Cre-p38γ flox/flox
D
Active B cells
Active T cells
Active CD4+ T cells
Active CD8+ T cells
CD19+ CD69+ cells (x10^6)
CD3+ CD69+ cells (x10^6)
CD3+ CD4+ CD69+ cells (x10^6)
CD3+ CD8+ CD69+ cells (x10^6)
p38δ flox/flox
CD4Cre-p38δ flox/flox
ns
Days
0
7
28

Suppl. Fig. 3

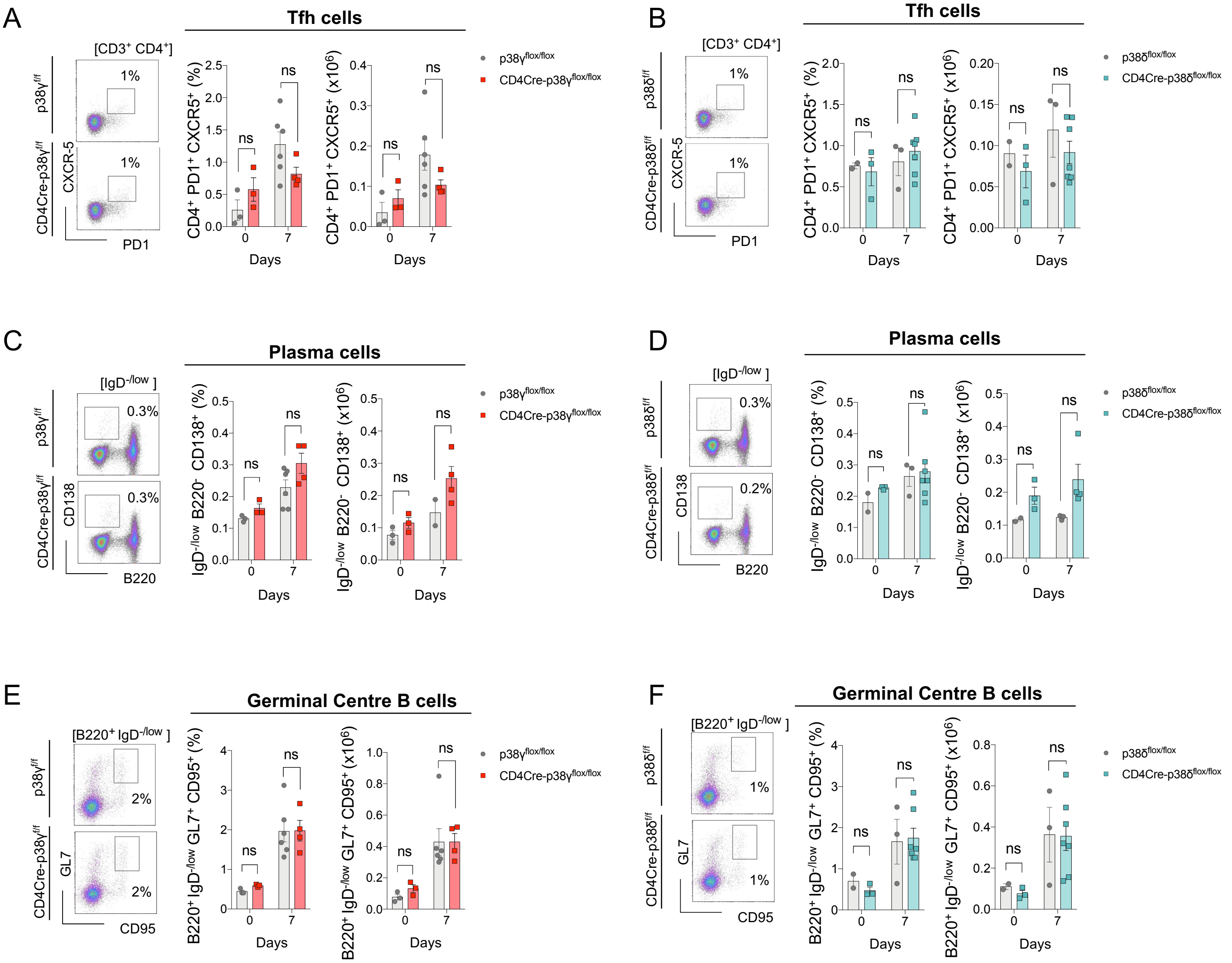
A
Tfh cells
[CD3+ CD4+]
p38γf/f
CD4Cre-p38γf/f
CXCR-5
PD1
1%
1%
CD4+ PD1+ CXCR5+ (%)
CD4+ PD1+ CXCR5+ (x10^6)
ns
Days
p38γflox/flox
CD4Cre-p38γflox/flox
B
Tfh cells
[CD3+ CD4+]
p38δf/f
CD4Cre-p38δf/f
p38δflox/flox
CD4Cre-p38δflox/flox
C
Plasma cells
[IgD-/low]
CD138
B220
0.3%
0.2%
IgD-/low B220- CD138+ (%)
IgD-/low B220- CD138+ (x10^6)
D
Plasma cells
E
Germinal Centre B cells
[B220+ IgD-/low]
GL7
CD95
2%
B220+ IgD-/low GL7+ CD95+ (%)
B220+ IgD-/low GL7+ CD95+ (x10^6)
F
Germinal Centre B cells

Suppl. Fig. 4
