# Supplementary material for: p38γ and p38δ support CD4+ T cell responses and T cell-dependent humoral immunity
Source: Front Immunol. 2026 Aug 26;17:1927358. doi: 10.3389/fimmu.2026.1927358 (PMC13553806; doi:10.3389/fimmu.2026.1927358)
Supplement: Supplementary file 2 [file Table1.docx]

**Table 1. Primers used for mice genotyping.**

| **Gene** | **Primer sequence (5’ – 3’)** |
| --- | --- |
| CD4-Cre | GTTCTTTGTATATATTGAATGTTAGCC |
|  | TATGCTCTAAGGACAAGAATTGACA |
|  | CTTTGCAGAGGGCTAACAGC |
| Mapk12-flox | CTGAGGCGGAAAGAACCAGCTG |
|  | CCAGGAGGTGACCAAAACGGC |
|  | TGGGCTCGCAAGGTAGAGGTG |
| Mapk13-flox | GAGCTACCAAGACCTACCTG |
|  | GGATGTGGAATGTGTGCGAGG |
|  | GCTCAGCTTCTTGATGGCCAC |

**Table 2. Antibodies used for flow cytometry analysis**

| **Antibody** | **Manufacturer** | **Reference** | **Concentration** | **Dilution** | **Organ** |
| --- | --- | --- | --- | --- | --- |
| CD3-Pecf594 | eBioscience | 562332 | 0,2 mg/ml | 1/200 | T |
| CD4-PercpCy5.5 | BioLegend | 100434 | 0,2 mg/ml | 1/200 | T |
| CD8-ef450 | eBioscience | 48-0081-82 | 0,05 mg/ml | 1/100 | T |
| CD25-PE | Pharmigen | 553866 | 0,2 mg/ml | 1/100 | T |
| CD44-FITC | SouthernBiotech | 1500-02 | 0,5 mg/ml | 1/50 | T |
| CD4-APCef780 | eBioscience | 47-0042-82 | 0,2 mg/ml | 1/200 | LN |
| CD8-PERCP | BioLegend | 100732 | 0,025 mg/ml | 1/100 | LN |
| B220-Pacific Blue | eBioscience | 48-0452-82 | 0,2 mg/ml | 1/100 | S |
| CD3-APC-eFluor780 | eBioscience | 47-0032-82 | 0,2 mg/ml | 1/50 | S |
| CD3-FITC | B.Coulter | 731979 | 0,5 mg/ml | 1/100 | S |
| CD4-PERCPCy5.5 | BioLegend | 100434 | 0,2 mg/ml | 1/100 | S |
| CD8-eFluor450 | eBioscience | 48-0081-82 | 0,05 mg/ml | 1/100 | S |
| CD19-AlexaFluor700 | eBioscience | 56-0193-82 | 0,05 mg/ml | 1/100 | S |
| CD44-PE | SouthernBiotech | 1500-09L | 0,2mg/ml | 1/50 | S |
| CD62L-APC | eBioscience | 17-0621-83 | 0,025 mg/ml | 1/100 | S |
| CD69-PECy7 | Invitrogen | 25-0691-82 | 0,2 mg/ml | 1/200 | S |
| CD95-PE | Pharmigen | 554258 | 0,2 mg/ml | 1/100 | S |
| CD138-PE | BioLegend | 142504 | 0,2 mg/ml | 1/200 | S |
| CD279-APC-eFluor780 | eBioscience | 47998582 | 0,2mg/ml | 1/50 | S |
| CXCR5-PE | BioLegend | 145504 | 0,2 mg/ml | 1/200 | S |
| IgD-FITC | B.Coulter | 732388 | 0,125 mg/ml | 1/100 | S |
| GL7-Biotin | eBioscience | 13-5902-85 | 0,5 mg/ml | 1/100 | S |
| Streptavidin-PECy7 | BioLegend | 405206 | 0,2 mg/ml | 1/200 | S |
| Streptavidin-PE | B.Coulter | 732998 | 0,125 mg/ml | 1/100 | S |
| CD23-Biotin | BD | 553137 | 0,5 mg/ml | 1/50 | S |
| CD21-FITC | BD | 553818 | 0,5 mg/ml | 1/200 | S |

T: Thymus; LN: Lymph node; S: Spleen.

**Table 3. Primers used for RT-qPCR**

| **Gene** | **Sense** | **Primer sequence (5’ – 3’)** |
| --- | --- | --- |
| Actb | Forward | AAGGAGATTACTTGCTCTGGCTCCTA |
|  | Reverse | ACTCATCGTACTCCTGCTTGCTGAT |
| Cxcl13 | Forward | CATAGATCGGATTCAAGTTACGCC |
|  | Reverse | TCTTGCTCCAGATCACAACTTCA |
| Il4 | Forward | CATCGGCATTTTGAACGAG |
|  | Reverse | CGAGCTCACTCTCTGTGGTG |
| Il10 | Forward | CAGGACTTTAAGGGTTACTTG |
|  | Reverse | ATTTTCACAGGGGAGAAATC |
| Il17f | Forward | CCCATGGGATTACAACATCACTC |
|  | Reverse | CACTGGGCCTCAGCGATC |
| Il21 | Forward | TCATCATTGACCTCGTGGCCC |
|  | Reverse | ATCGTACTTCTCCACTTGCAATCCC |
| Mapk12 | Forward | ACCTGATGAGTCTCTGGACGA |
|  | Reverse | CAGATCAGTGCCCATGAAT |
| Mapk13 | Forward | GGACCCTGAGGAGGAGACA |
|  | Reverse | GTTTGAGATCTCTTTGTAGATGTGTTG |
